# Pediatric antibiotic stewardship programs in Europe: a pilot survey among delegates of The European Academy of Pediatrics

**DOI:** 10.3389/fped.2023.1157542

**Published:** 2023-06-05

**Authors:** Stephen M. Reingold, Zachi Grossman, Adamos Hadjipanayis, Stefano Del Torso, Arunas Valiulis, Lukasz Dembinski, Shai Ashkenazi

**Affiliations:** ^1^ Department of Pediatrics, Meuhedet Health Maintenance Organization, Tel Aviv, Israel; ^2^ Adelson School of Medicine, Ariel University, Ariel, Israel; ^3^ Department of Pediatrics, Maccabi Health Services, Tel Aviv, Israel; ^4^ Medical School, European University Cyprus, Nicosia, Cyprus; ^5^ Department of Paediatrics, Larnaca General Hospital, Larnaca, Cyprus; ^6^ Department of Pediatrics, ChildCare WorldWide—CCWWItalia OdV, Padova, Italy; ^7^ Clinic of Children’s Diseases, Institute of Clinical Medicine, Medical Faculty of Vilnius University, Vilnius, Lithuania; ^8^ Human Ecology Research Group, Department of Public Health, Institute of Health Sciences, Medical Faculty of Vilnius University, Vilnius, Lithuania; ^9^ Department of Pediatric Gastroenterology and Nutrition, Medical University of Warsaw, Warsaw, Poland

**Keywords:** antibiotic resistance, antibiotic stewardship, Europe, infectious diseases, pediatrics, antimicrobial resistance (AMR)

## Abstract

**Background:**

Antimicrobial resistance (AMR) is one of the leading causes of morbidity and mortality worldwide. Efforts to promote the judicious use of antibiotics and contain AMR are a priority of several medical organizations, including the WHO. One effective way to achieve this goal is the deployment of antibiotic stewardship programs (ASPs). This study aimed to survey the current situation of pediatric ASPs in European countries and establish a baseline for future attempts to harmonize pediatric ASPs and antibiotic use in Europe.

**Methods:**

A web-based survey was conducted among national delegates of the European Academy of Paediatrics (EAP). The survey assessed the presence of pediatric ASPs in the representatives’ countries in the inpatient and outpatient settings, the staff included in the programs, and their detailed activities regarding antibiotic use.

**Results:**

Of the 41 EAP delegates surveyed, 27 (66%) responded. Inpatient pediatric ASPs were reported in 74% (20/27) countries, and outpatient programs in 48% (13/27), with considerable variability in their composition and activities. Guidelines for managing pediatric infectious diseases were available in nearly all countries (96%), with those for neonatal infections (96%), pneumonia (93%), urinary tract (89%), peri-operative (82%), and soft tissue (70%) infections being the most common. Pediatric ASPs were reported at the national (63%), institutional (41%), and regional/local (<15%) levels. Pediatricians with infectious disease training (62%) and microbiologists (58%) were the most common members of the program personnel, followed by physician leaders (46%), infectious disease/infection control physicians (39%), pharmacists (31%), and medical director representatives (15%). Activities of the pediatric ASPs included educational programs (85%), monitoring and reporting of antibiotic use (70%) and resistance (67%), periodic audits with feedback (44%), prior approval (44%), and post-prescription review of selected antibiotic agents (33%).

**Conclusion:**

Although pediatric ASPs exist in most European countries, their composition and activities vary considerably across countries. Initiatives to harmonize comprehensive pediatric ASPs across Europe are needed.

## Introduction

The development and wide use of antibiotic agents have revolutionized the physician's ability to effectively treat bacterial infections and significantly reduce the morbidity and mortality caused by these infections (1). The evolution and spread of bacteria resistant to antibiotic agents endanger these medical achievements (2, 3). A recent systematic analysis of the global burden of antimicrobial resistance (AMR) in 204 countries concluded that AMR was associated with and attributed to 4.95 and 1.27 million annual deaths, respectively (2). A European study found gaps in healthcare workers’ knowledge and behavior regarding antibiotics and AMR (4), highlighting the need for efforts to promote appropriate antibiotic use in Europe (5). The World Health Organization (WHO) declared in 2021 that “AMR is one of the top 10 global public health threats facing humanity” (3). Alarms of a possible entrance into a post-antibiotic era have been raised (6).

The main drivers of AMR are the overuse and misuse of antibiotics, mainly by inducing a selective pressure (1, 3, 6), reflecting the need for global actions to control antibiotic prescribing (6, 7). Antibiotic consumption also has a prolonged influence on the intestinal microbiota, with increased risks of various medical conditions, such as diabetes, obesity, and inflammatory bowel disease (8). Moreover, antibiotic overuse in humans affects our planet, including its animals, soil, rivers, and aquatic wildlife, endangering our future ability to effectively treat bacterial infections as well as the sustainability of our planet (9).

Antibiotic stewardship is defined as continuing activities by healthcare personnel to optimize clinical antimicrobial use, namely, prescribing antibiotics when needed, using the most appropriate antimicrobial agent, the right dose, at the right time, and for the needed duration (10, 11). Pediatric antibiotic stewardship programs (ASPs) include designated teams that coordinate interventions and educational activities to monitor and improve antibiotic use in children, at the local, regional, national, and international levels (10, 12–14). Individual studies and meta-analyses have demonstrated the implementation, outcome, and constituency of pediatric ASPs worldwide (15, 16). Attempts to establish pediatric ASPs in Europe have been documented (10, 14). Others have surveyed pediatric ASPs, but only in individual European countries (17, 18).

The present study aimed to survey pediatric ASPs in European countries in the inpatient and outpatient settings, and to analyze their composition and activities, with the attempt to build a baseline for future harmonization in Europe.

## Materials and methods

### Study design and data collection

The survey was conducted among the national delegate pediatricians of 41 countries in Europe and its surroundings that are represented in the European Academy of Paediatrics (EAP). These countries include members of the European Union, the European Economic Area and Associate Countries, according to the EAP Article of Association. The delegates are nominated by the respective national professional and/or scientific organization. Participation in the survey was voluntary. As emphasized in the survey invitation, delegates could appoint a local committee member to reply and/or to consult with other stakeholders in their country (for example, national leaders in pediatric infectious diseases or those involved in antibiotic stewardship activities at the national level) before responding to the questionnaire. The survey was approved by the Board of the EAP, distributed in May 2022, and two reminders were sent in June 2022. No incentive was given. Data were collected online, and descriptive analysis was performed. For the purposes of data analysis, multiple answers from a country were collated into a single response for that country.

For all questions, a positive response overrode a negative response within an individual country. For binary questions, such as the presence of a national pediatric ASP, an affirmative response from any responder of that country resulted in an affirmative collated response for that country. For multiple variable questions (such as the components of a pediatric ASP), all selected components for all the responders of a given country were included.

### Study questionnaire

The study questionnaire had eight questions organized into five sections (see Supplementary Table S1):

1. Identifiers of the city and country of the responder.
2. Existing formal inpatient and outpatient pediatric ASPs in the country.
3. Availability of guidelines for diagnosing and treating infection syndromes in children (common and uncommon), hospital-acquired infections and for preventing surgical infections.
4. The detailed personnel in the inpatient pediatric ASPs.
5. The detailed interventions used by the pediatric ASPs.

## Results

### Respondent characteristics

Representatives of 27/41 countries responded (response rate 66%). Several country representatives shared the questionnaire with local experts or committee members, resulting in multiple responses for six of the 27 countries that replied.

The countries that responded and were included in the survey (with the number of >1 respondents in parenthesis) were: Armenia, Austria, Belgium (3), Croatia, Cyprus (4), Czech Republic, Estonia, Finland, France, Germany, Ireland, Israel, Italy (2), Lithuania, Luxembourg, Malta, North Macedonia (2), Norway, Poland, Portugal (2), Republic of Bosnia and Herzegovina, Serbia, Slovenia, Spain (8), Sweden, Switzerland, and Ukraine.

### Pediatric ASPs in inpatient and outpatient settings

The national delegates reported that in 20 (74%) countries, pediatric ASPs for inpatient medical facilities (mainly hospitals) did exist; in 6 (22%) countries, there were no programs in these settings. Regarding outpatient medical facilities, pediatric ASPs were present in only 13 (48%) of the countries, with 5 (19%) of the national delegates being unsure.

Availability of clinical practice guidelines

Guidelines for the diagnosis and management of pediatric infectious syndromes were reported as available in nearly all countries, namely 26/27 (96%) of the responding delegates. In most countries (63%) the guidelines were national, followed by institutional guidelines in 41%, and only a minority having regional (4; 15%) or local (1; 4%) departmental guidelines.

Guidelines for common infectious syndromes and diseases in children were reported in most countries. For example, protocols for the approach and management of suspected neonatal infections (96%), community-acquired pneumonia (93%), urinary tract infections (89%), and skin and soft tissue infections (70%). Protocols for peri-operative antibiotic prophylaxis were also common (82%); unfortunately, protocols for less common infectious syndromes, such as osteo-articular infections, and for hospital-related infections, were infrequently found (4% each).

### Personnel in the pediatric ASPs

The detailed recommendations for personnel are available mainly for inpatient pediatric ASPs; medical staff involved in inpatient European programs at the national level are shown in Table 1. In more than half of the responding countries, a pediatrician with formal infectious disease training and a representative of the microbiology laboratory were members of the pediatric ASP (62% and 58%, respectively). In contrast, an infectious disease or infection control specialist was present in 39% of the programs, a pharmacist in 31%, and a medical director or his representative only in 15%. A physician was the leader of the pediatric ASP in nearly half of the countries. There was substantial variability in ASPs composition across countries.

**Table 1:** Personnel included in the inpatient pediatric antibiotic stewardship programs among 26 European countries.

Personnel	Number	%
Physician Leader	12	46
Pediatrician with formal infectious disease training	16	62
Infectious disease/infection control physician	10	39
Pharmacist	8	31
Microbiology laboratory representative	15	58
Medical director representative	4	15

### Interventions used by the pediatric ASPs

Educational sessions on the judicious use of antibiotics were the most common (85%) intervention used by the pediatric ASPs to improve antibiotic prescription in the hospitals, followed by monitoring and reporting of antibiotic use in the various hospital departments by 70%, and periodic monitoring and reporting of AMR patterns by 67% of the programs. Regarding direct control of antibiotic use: in nearly half of the countries, pediatric ASPs performed prior approval of selected antimicrobial agents and conducted periodic audits with feedback to the hospital staff, and about one-third performed a post-prescription review within 48–72 h (Table 2).

**Table 2:** Interventions used by the pediatric antibiotic stewardship programs to improve antibiotic use in 27 European countries.

Intervention	Number	%
Prior approval of selected antibiotics	12	44
Post-prescription review (48–72 h)	9	33
Periodic audits with feedback	12	44
Monitoring antibiotic use and reporting	19	70
Monitoring antibiotic resistance and periodic reporting	18	67
Education sessions on the judicious use of antibiotics	23	85
None	2	7

## Discussion

The present study constitutes a pilot survey of pediatric ASPs in Europe and identifies substantial variability in the composition and activities of the surveyed ASPs.

Kopsidas et al. previously surveyed the presence of pediatric ASPs in 23 European countries (19). They also found significant variability and lack of consistency across the continent, with 33% of the respondents being unable to provide information on the availability of ASP programs within individual hospitals in their country. We report national guidelines on infections commonly seen in children in 64% of the countries surveyed, similar to the 70% reported by Kopsidas et al. The present study differs in several aspects. First, we sent questionnaires to national delegates of 41 country members of the EAP, with a response from 27. In addition, the present study details the presence of pediatric ASP guidelines within individual countries, inpatient pediatric ASP personnel and interventions, as well as the specific infectious diseases addressed.

Antibiotic-resistant bacteria know no borders, and therefore AMR cannot be tackled by a single country or institution; harmonization and cooperation among countries and regional alliances are crucial to successfully fight AMR (2, 5, 6). Progress has been made in adopting several essential surveillance systems across European countries, including antibiotic consumption, rates of AMR, antimicrobial susceptibility testing, and defined breakpoints (5, 10). These activities are important but insufficient, as AMR increases in Europe and globally (2, 10).

Pediatric ASPs varied in hierarchical level, among departmental, hospital, local, regional, or national levels. Not addressed in this study is how higher-level programs (such as the national and regional scale) are applied or implemented at the lower level (hospital or department), nor does it address possible conflicts between the programs regarding overlapping and conflicting activities. Guidelines were most common for the diagnosis and management of neonatal infections, but less common for peri-operative surgical management. The present study shows that some common serious bacterial infections are more likely to be addressed by pediatric ASPs than others (pneumonia and urinary tract infections, versus soft tissue, skin, and osteo-articular infections).

There is significant variability regarding the personnel involved in pediatric ASPs, as well as the way the programs are implemented. Pediatric ASPs in general are difficult to manage in real time when emergent situations arise. However, some protocols take such eventualities into account, while mitigating the prevalence of antibiotic overuse, utilizing protocols of antibiotic choice, emergent access to authorizing personnel and expert opinions, and multidisciplinary teams. Regular learning processes, such as case reviews, periodic meetings and interdisciplinary rounds all help to increase knowledge, improve practice, and avoid antibiotic misuse while learning from previous experiences (13). As pediatric ASPs are not consistently present nor coordinated in proximal regions, the benefit of such programs has not yet been fully realized. Creating coordinated pediatric ASPs from the department to the national level, with international feedback and cooperation, may potentially have a more significant impact.

This study has several limitations. The questionnaire was relatively short (in order to increase the response rate); thus, we consider our study as a pilot survey. The survey was not completed by all national delegates of the countries included in the EAP, which may have resulted in a selection bias, although the response rate of 66% is relatively high. The survey was conducted by questionnaires that had been sent to national delegates, who could consult with pediatric infectious diseases leaders and/or those involved in antibiotic stewardship activities at the national level, but we had no ability to verify the answers or to determine the actual practices and interventions in the respective countries. For several countries, we collated answers from multiple responders. It should be emphasized that within-country variability obviously exists and may not have been captured by our survey. Multiple choice answers limited options that may not have considered all possibilities, although the ability to add a free text was given. Because of these limitations, we consider our study a pilot survey; more detailed surveys, as was reported, for example, from the United Kingdom (17), are required.

## Conclusions

Pediatric ASPs are essential in ensuring appropriate antibiotic prescription, reducing antibiotic overuse and selective pressure, and avoiding unnecessary healthcare costs. However, their implementation in Europe is currently inconsistent and not unified: the survey demonstrates wide variability in the presence of pediatric ASPs across the continent, as well as in their personnel and activities. More detailed and comprehensive surveys are needed to confirm these findings, as well as initiatives to comprehensively harmonize pediatric ASPs across the European continent.

## Data Availability

The raw data supporting the conclusions of this article will be made available by the authors, without undue reservation.
