# Supplementary material for: Pediatric antibiotic stewardship programs in Europe: a pilot survey among delegates of The European Academy of Pediatrics
Source: Front Pediatr. 2023 Jun 5;11:1157542. doi: 10.3389/fped.2023.1157542 (PMC10277725; doi:10.3389/fped.2023.1157542)
Supplement: Supplementary file 1 [file Datasheet1.pdf]

Supplement Table 1: Questionnaire

1. What country do you represent?
2. What is your city?
3. Is there an ASP in your country for inpatient setting?
4. Is there an ASP in your country for outpatient setting?
5. Do you have guidelines (national, regional, local, or institutional) for diagnosing & treating common infection syndromes or for preventing surgical infections, such as (include all that apply):
    a. None
    b. Community-acquired pneumonia
    c. Urinary tract infection
    d. Skin and soft-tissue infections
    e. Neonatal infections
    f. Peri-operative antibiotic prophylaxis
    g. Don’t know / unsure
    h. Other:
6. Are these guidelines (please check all that apply):
    a. National
    b. Regional
    c. Local
    d. Institutional
    e. Don’t know / unsure
    f. Other:
7. Please indicate the personnel in the inpatient ASP (include all that apply):
    a. None
    b. Physician leader
    c. Pediatrician with formal infectious disease training
    d. Pharmacist
    e. Infection control physician
    f. Microbiology laboratory representative
    g. Medical center director representative
    h. Don’t know / unsure
    i. Other:

8. Please indicate the intervention used by your ASP to improve antibiotic use (include all that apply):
    a. None
    b. Prior approval of selected antibiotics
    c. Post-prescription (48-72h) review of antibiotics
    d. Periodic audits with feedback

e. Monitoring antibiotic use and reporting
f. Monitoring antibiotic resistance and periodic reporting
g. Education sessions on judicious antibiotic use
h. Don’t know / unsure
i. Other:
